# Effectiveness of Cognitive Rehabilitation in Parkinson’s Disease: A Systematic Review and Meta-Analysis

**DOI:** 10.3390/jpm11050429

**Published:** 2021-05-18

**Authors:** Itsasne Sanchez-Luengos, Yolanda Balboa-Bandeira, Olaia Lucas-Jiménez, Natalia Ojeda, Javier Peña, Naroa Ibarretxe-Bilbao

**Affiliations:** Department of Methods and Experimental Psychology, Faculty of Psychology and Education, University of Deusto, 48007 Bilbao, Spain; itsasnesanchez@deusto.es (I.S.-L.); yolandabalboa@deusto.es (Y.B.-B.); olaia.lucas@deusto.es (O.L.-J.); nojeda@deusto.es (N.O.); javier.pena@deusto.es (J.P.)

**Keywords:** Parkinson’s disease (PD), cognitive rehabilitation, intervention, cognition

## Abstract

Cognitive deficits influence the quality of life of Parkinson’s disease (PD) patients. In order to reduce the impact of cognitive impairment in PD, cognitive rehabilitation programs have been developed. This study presents a systematic review and meta-analysis regarding the effectiveness of cognitive rehabilitation in non-demented PD patients. Twelve articles were selected according to PRISMA guidelines. The systematic review showed that attention, working memory, verbal memory, executive functions and processing speed were the most frequently improved domains. Meta-analysis results showed moderate effects on global cognitive status (*g* = 0.55) and working memory (*g* = 0.50); small significant effects on verbal memory (*g* = 0.41), overall cognitive functions (*g* = 0.39) and executive functions (*g* = 0.30); small non-significant effects on attention (*g* = 0.36), visual memory (*g* = 0.29), verbal fluency (*g* = 0.27) and processing speed (*g* = 0.24); and no effect on visuospatial and visuoconstructive abilities (*g* = 0.17). Depressive symptoms showed small effect (*g* = 0.24) and quality of life showed no effect (*g* = −0.07). A meta-regression was performed to examine moderating variables of overall cognitive function effects, although moderators did not explain the heterogeneity of the improvement after cognitive rehabilitation. The findings suggest that cognitive rehabilitation may be beneficial in improving cognition in non-demented PD patients, although further studies are needed to obtain more robust effects.

## 1. Introduction

Parkinson’s disease (PD) is the second most common neurodegenerative disease after Alzheimer’s disease [1]. PD is associated with motor (bradykinesia, rigidity and tremor) and non-motor symptoms such as cognitive impairment, neuropsychiatric symptoms, sensory abnormalities, sleep disorders and autonomic disturbances [1,2].

Cognitive deficits are usually associated with PD [3], with 40% of PD patients developing mild cognitive impairment (MCI) during the course of the disease [4], and with attention, executive functions, visuospatial abilities and memory being the most affected domains [5,6]. In turn, the risk of dementia increases with the deterioration of cognitive deficits and disease progression [7], with 83% of PD patients with cognitive impairment presenting dementia after 20 years [8].

Non-pharmacological interventions have been developed [9] with the aim of intervening on the cognitive and functional impairment of PD, with cognitive rehabilitation being one of the strategies suggested for personalized medicine [10]. Several systematic reviews [11,12,13,14,15,16] and meta-analyses [17,18,19] have reviewed and analyzed the effect of cognitive rehabilitation in PD, suggesting that this intervention may be potentially beneficial in increasing cognitive performance or maintaining cognitive levels over time, especially when treatment is applied before dementia has set in [12]. As far as the authors are aware, there have only been three meta-analyses that analyze the effectiveness of cognitive rehabilitation in PD to date. Specifically, Leung and colleagues conducted the first meta-analysis published in 2015 [18], focused on the analysis of seven randomized controlled trials (RCTs), in which they showed improvements in working memory, executive functions and processing speed. Later, in 2017, Lawrence and colleagues [17] examined the effectiveness of cognitive rehabilitation along with non-invasive brain stimulation interventions, and found improvements in attention/working memory, memory and executive functions. Orgeta and colleagues [19] published the latest meta-analysis in 2020, which covered the effects of cognitive rehabilitation from seven RCTs in PD patients with MCI or dementia (excluding PD patients without MCI or dementia), and found no evidence of cognitive improvement after cognitive rehabilitation [19].

It is important to emphasize that there is an increasing number of studies that examine the effects of cognitive rehabilitation in PD patients without dementia. Therefore, it is essential to conduct an updated review of the literature to include and analyze the effect sizes that have been published to date. In addition, variables related to the characteristics and progression of the disease could interfere on the benefit of rehabilitation in people with PD [20]. The possible influence of rehabilitation characteristics (modality (paper/pencil or computer-based exercises), the duration of the entire program, and the frequency and duration of the sessions) should also be investigated. However, none of the meta-analyses published in PD performed moderator analyses to analyze the existence of possible factors that may influence the cognitive rehabilitation process. Therefore, the aim of this systematic review and meta-analysis was to conduct a critical review of the effectiveness of cognitive rehabilitation in PD and to analyze whether cognitive rehabilitation improves cognition, functionality, depressive symptoms and quality of life in people with PD. In addition, we analyzed the influence of age and years of education, variables related to intervention, baseline global cognitive scores and PD patient-related features on the effectiveness of cognitive rehabilitation in PD.

## 2. Materials and Methods

### 2.1. Search Strategy

A systematic review and meta-analysis of published research papers that focus on cognitive rehabilitation in PD patients was conducted according to the guidelines of “Preferred Information Elements for Systematic Reviews and Meta-Analysis” (PRISMA) [21]. This systematic review protocol was registered in the International Prospective Register of Systematic Reviews, PROSPERO (CRD42021243716). The bibliographic search was conducted in December 2020 and April 2021. PubMed database was used and the specific terms used for searching, identifying and selecting studies were: (1) Parkinson’s disease and Parkinson disease; (2) cognitive rehabilitation/training/remediation/stimulation; (3) attention; (4) working memory; (5) memory; (6) executive functions and (7) rehabilitation/training/remediation/stimulation. The terms were combined in order to conduct a more comprehensive search: (1) + (2); (1) + (3) + (7); (1) + (4) + (7); (1) + (5) + (7); and (1) + (6) + (7). The search was filtered by title or abstract. In addition, we performed a citation search of the reference lists from the meta-analyses [17,18,19] of cognitive rehabilitation in PD published to date. For a detailed description of the search strategy applied and the number of results obtained, see Supplementary Material Table S1.

### 2.2. Eligibility Criteria

The inclusion criteria for studies were: (1) patients with a diagnosis of idiopathic PD, (2) PD patients receiving structured cognitive rehabilitation, (3) analysis of the effects on cognition and (if included in the studies) on functionality, depression and quality of life, (4) study design (parallel controlled trials), (5) studies that compared structured cognitive intervention with a control group receiving no specific cognitive intervention or unstructured cognitive intervention and (6) studies assessing outcomes immediately after the intervention period. As for the exclusion criteria, these were: (1) review papers, (2) single case studies, (3) conference abstract or presentation, (4) PD patients diagnosed with dementia, (5) brain stimulation studies, (6) only neuroimaging data, (7) lack of available data for effect size estimation, (8) lack of PD control group and (9) article language, as those written in any language other than English were excluded.

### 2.3. Quality Assessment and Risk of Bias in the Included Studies

The risk of bias in the included studies was assessed using the Cochrane Manual for Systematic Reviews of Interventions for RCTs (RoB 2 tool) [22] and non-randomized trials (ROBINS-I tool) [23]. This tool evaluated different aspects of the trial design, conduct and report, in order to obtain information about the characteristics of the trial relevant to the risk of bias [24]. Moreover, the methodological quality of the randomized studies was assessed using the Physiotherapy Evidence Database (PEDro-P) Rating Scale [25], comprising 11 items. For this purpose, the evaluation of the quality and risk of bias of the studies was evaluated independently by two reviewers (I.S.-L. and Y.B.-B.), obtaining an inter-rater reliability of 83.56%. The risk of bias was classified as low, unclear or high (See Table S2).

### 2.4. Data Extraction

Specific information was extracted for the systematic review from the studies selected, which included: (1) first author and year of publication; (2) sample size of the study; (3) characteristics of the sample; (4) characteristics of the disease; (5) type of intervention; (6) format of the intervention; (7) duration of the intervention; (8) cognitive domains trained and (9) variables in which improvements have been obtained after cognitive rehabilitation. In the meta-analysis, all effect sizes were calculated from the means and standard deviations, and/or *F* scores. Effect sizes were calculated for overall cognitive functions, global cognitive status and eight specific cognitive subdomains: attention, working memory, verbal memory, visual memory, verbal fluency, executive functions, visuospatial and visuoconstructive abilities, and processing speed. The effect sizes of the overall cognitive functions were calculated based on the mean of the global cognitive status and cognitive subdomains obtained from this meta-analysis. For this purpose, studies included in this meta-analysis that had reported at least two cognitive domains were selected. Moreover, the effect sizes of depressive symptoms and quality of life were also calculated. The tests included were classified according to the domain assessed by the test itself (See Table S3). For the moderator or meta-regression analyses, the following were selected: specific scores of the age and years of education, variables related to intervention time, the modality of delivering the cognitive training (pencil and paper or computer), baseline global cognitive scores and PD patient-related features (disease duration, the Unified Parkinson’s Disease Rating Scale (UPDRS) part III [26] and the Hoehn and Yahr scale (H&Y) [27]).

### 2.5. Statistical Analysis

In this meta-analysis, we estimated the effect sizes of 12 articles in order to analyze the differences in the effects of cognitive rehabilitation programs on PD patients compared to control groups. We calculated the standardized mean difference (SMD), using Cohen’s *d* formula [28] first, to estimate all the different outcomes. SMD values were calculated with a 95% confidence interval (CI) from the means and standard deviations, and the *F* scores provided by the studies, and were corrected using Hedges’ *g* small sample size bias adjustment formula [29]. For those studies that provided the necessary data, the change score to estimate the effects sizes was calculated based on the assumption that the correlation between measures at pretest and posttest times is zero. This is a conservative approach that was previously used in Orgeta and colleagues’ meta-analysis [19]. A random-effects model was used to perform all meta-analysis estimations. We did not detect or remove any effect size as an outlier. Effect sizes were considered small ≥0.20, moderate ≥0.50, or large ≥0.80. Heterogeneity across the studies was estimated using Cochrane’s Q test (Q) and *I^2^* indexes, where the *I^2^* index can indicate low (25%), medium (50%), or high (75%) heterogeneity [30].

We examined the possible influence of different predictor variables on the effect sizes obtained in overall cognitive functions, using multiple meta-regression analyses with the rma function and the dmetar package available in RStudio [31]. These moderator analyses were conducted using the mixed-effects model [32].

Publication bias refers to the tendency to submit or accept articles for publication based only on the positive findings [33], and is a concern in meta-analyses as it can influence the validity of the analyses conducted [34]. Therefore, publication bias was assessed for the different studies using funnel plots and the Egger regression test [35]. Finally, sensitivity analyses were conducted to assess possible changes in the previously obtained results, with only RCTs included.

All the effect size calculations and analyses were conducted using the Practical Meta-Analysis Effect Size Calculator, Review Manager (RevMan, Version 5.4, Cochrane, London, UK), and the meta and metafor packages in RStudio (Version 1.3.1093, RStudio, PBC, Boston, MA, USA).

## 3. Results

### 3.1. Study Selection

**Figure 1:**
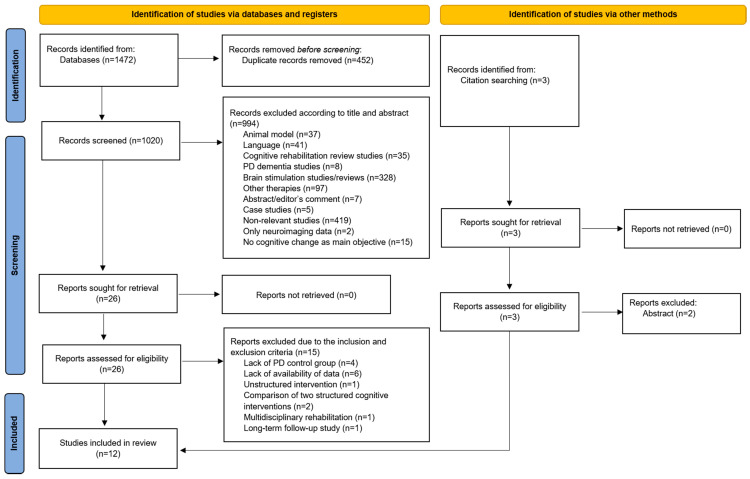
PRISMA summary of identified studies included in the review.

Initially, 1472 articles were identified in PubMed database. Four hundred and fifty-two articles were excluded because they were duplicates. From the remaining 1020 articles, 994 were removed following initial screening based on their title and abstract. Twenty-six articles were assessed for eligibility and 15 of those studies were excluded due to the inclusion and exclusion criteria. Additionally, three studies were selected by citation searching, of which two were excluded. Finally, 12 articles were selected for the systematic review and the meta-analysis (see Figure 1).

### 3.2. Study Characteristics

Twelve studies with a total of 512 participants with PD were included in the systematic review and meta-analysis. Selected studies were published between 2004 and 2020, with the number of participants involved in the studies ranging from 15 to 75. Eleven of the 12 studies found no differences at baseline in demographic variables, whereas one study showed significant differences in age, sex, and years of disease progression, so the subsequent analyses were adjusted. The disease stage score assessed by H&Y was between 1 and 3 in most studies. The frequency of the interventions ranged from twice a week to five times a week, with a maximum duration of 90 min, over a period of time that varied from three weeks to six months. Two different methods of intervention were used; four studies conducted cognitive rehabilitation activities using a pencil and paper format, and five studies with a computer-based format. Three studies also used both methods (pencil and paper and computer-based activities) in their cognitive rehabilitation sessions. Six studies conducted the intervention in a group format, while another study conducted the intervention individually. Most of the research focused on cognitive outcomes and reported improvements, although there was diversity in the number of cognitive domains showing improvement, ranging from a single domain to six. A summary of the studies included in cognitive rehabilitation for PD is shown in Table 1.

### 3.3. Effectiveness of Cognitive Rehabilitation

#### 3.3.1. Overall Cognitive Functions

**Figure 2:**
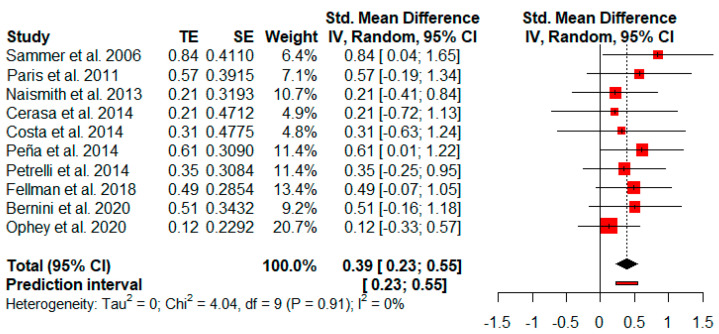
Effectiveness of cognitive rehabilitation in overall cognitive functions.

The effect size of overall cognitive functions was based on the mean of global cognitive status and cognitive subdomains from 10 studies reporting at least two cognitive variables. The random-effects model showed a small and statistically significant effect size (*g* = 0.39, *p* = 0.01) with a 95% CI (0.23 to 0.55). The heterogeneity test showed low levels of heterogeneity across the studies (Q = 4.04, *p* = 0.91; *I^2^* = 0%) (Figure 2).

#### 3.3.2. Global Cognitive Status

**Figure 3:**
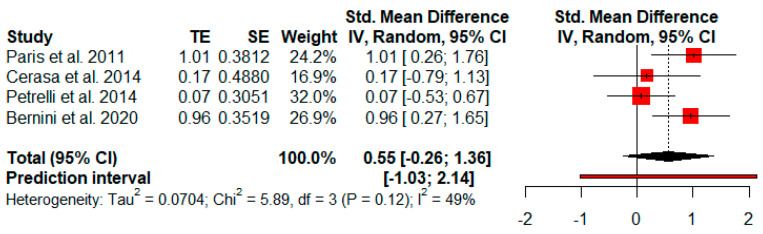
Effectiveness of cognitive rehabilitation in global cognitive status.

Four studies analyzed changes in global cognitive status after cognitive rehabilitation using global cognitive screening tests. The random-effects model showed a moderate and not significant effect size (*g* = 0.55, *p* = 0.12) with a 95% CI (−0.26 to 1.36). The heterogeneity test showed moderate levels of heterogeneity (Q = 5.89, *p* = 0.12; *I^2^* = 49%) (Figure 3).

#### 3.3.3. Attention

**Figure 4:**
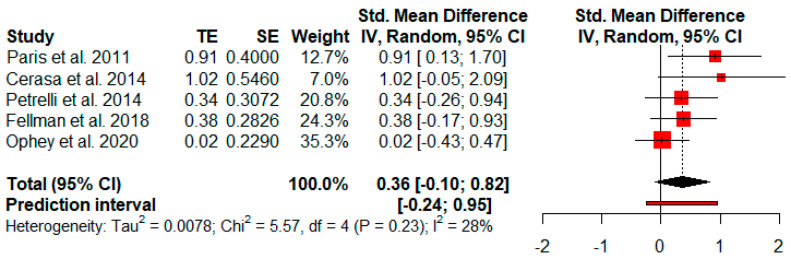
Effectiveness of cognitive rehabilitation in attention.

Five studies analyzed changes in attention after cognitive rehabilitation. The random-effects model showed a small and not significant effect size (*g* = 0.36, *p* = 0.09) with a 95% CI (−0.10 to 0.82). The heterogeneity test showed low levels of heterogeneity (Q = 5.57, *p* = 0.23; *I^2^* = 28%) (Figure 4).

#### 3.3.4. Working Memory

**Figure 5:**
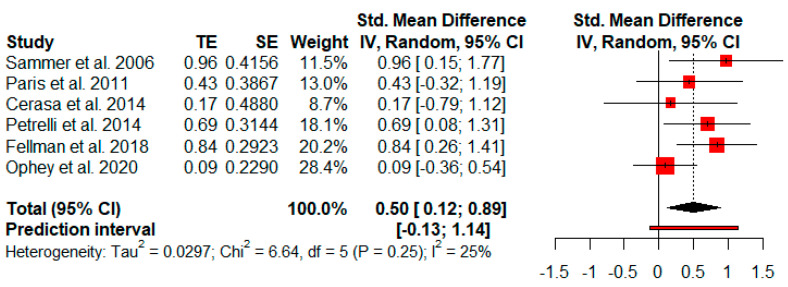
Effectiveness of cognitive rehabilitation in working memory.

Six studies analyzed changes in working memory after cognitive rehabilitation. The random-effects model showed a moderate and statistically significant effect size (*g* = 0.50, *p* = 0.02) with a 95% CI (0.12 to 0.89). The heterogeneity test showed low levels of heterogeneity (Q = 6.64, *p* = 0.25; *I*^2^ = 25%) (Figure 5).

#### 3.3.5. Verbal Memory

**Figure 6:**
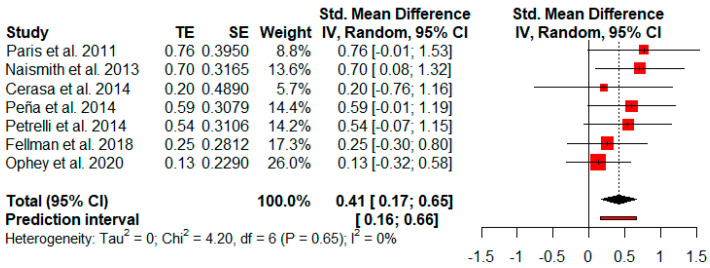
Effectiveness of cognitive rehabilitation in verbal memory.

Seven studies reported verbal memory measures after cognitive rehabilitation. The random-effects model showed a small and statistically significant effect size (*g* = 0.41, *p* = 0.00) with a 95% CI (0.17 to 0.65). The heterogeneity test showed low levels of heterogeneity (Q = 4.20, *p* = 0.65; *I*^2^ = 0%) (Figure 6).

#### 3.3.6. Visual Memory

**Figure 7:**
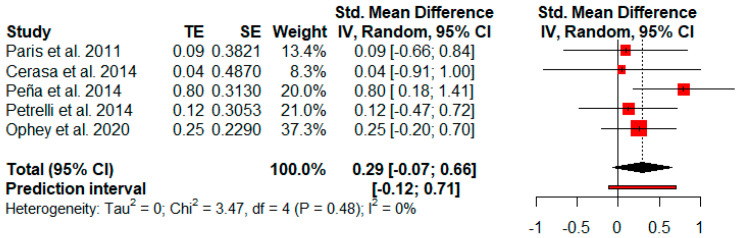
Efficacy of cognitive rehabilitation in visual memory.

Five studies reported visual memory measures after cognitive rehabilitation. The random-effects model showed a small and not significant effect size (*g* = 0.29, *p* = 0.08) with a 95% CI (−0.07 to 0.66). The heterogeneity test showed low levels of heterogeneity (Q = 3.47, *p* = 0.48; *I*^2^ = 0%) (Figure 7).

#### 3.3.7. Verbal Fluency

**Figure 8:**
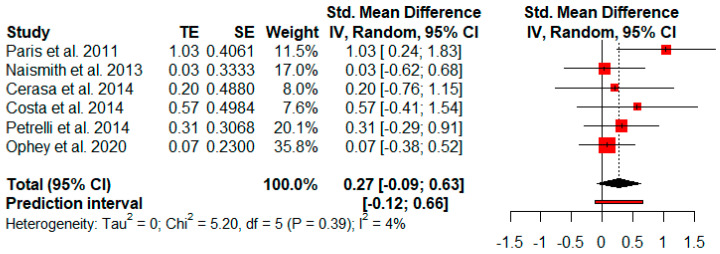
Effectiveness of cognitive rehabilitation in verbal fluency.

Six studies measured verbal fluency after cognitive rehabilitation. The random-effects model showed a small and not significant effect size (*g* = 0.27, *p* = 0.11) with a 95% CI (−0.09 to 0.63). The heterogeneity test showed low levels of heterogeneity (Q = 5.20, *p* = 0.39; *I*^2^ = 4%) (Figure 8).

#### 3.3.8. Executive Functions

**Figure 9:**
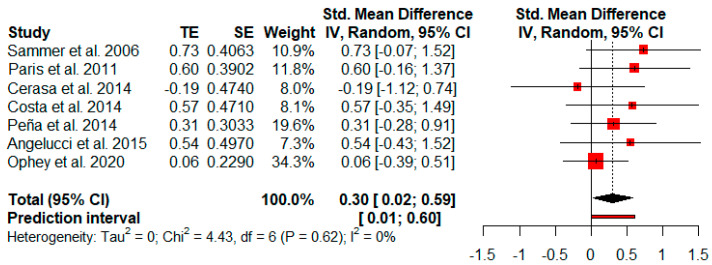
Effectiveness of cognitive rehabilitation in executive functions.

Seven studies reported executive function measures. The random-effects model showed a small and statistically significant effect size (*g* = 0.30, *p* = 0.04) with a 95% CI (0.02 to 0.59). The heterogeneity test showed low levels of heterogeneity (Q = 4.43, *p* = 0.62; *I*^2^ = 0%) (Figure 9).

#### 3.3.9. Visuospatial and Visuoconstructive Abilities

**Figure 10:**
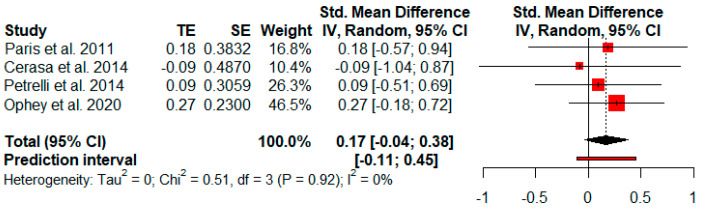
Effectiveness of cognitive rehabilitation in visuospatial and visuoconstructive abilities.

Four studies analyzed changes in visuospatial and visuoconstructive abilities after cognitive rehabilitation. The random-effects model showed no effect size (*g* = 0.17, *p* = 0.11) with a 95% CI (−0.04 to 0.38). The heterogeneity test showed low levels of heterogeneity (Q = 0.51, *p* = 0.92; *I*^2^ = 0%) (Figure 10).

#### 3.3.10. Processing Speed

**Figure 11:**
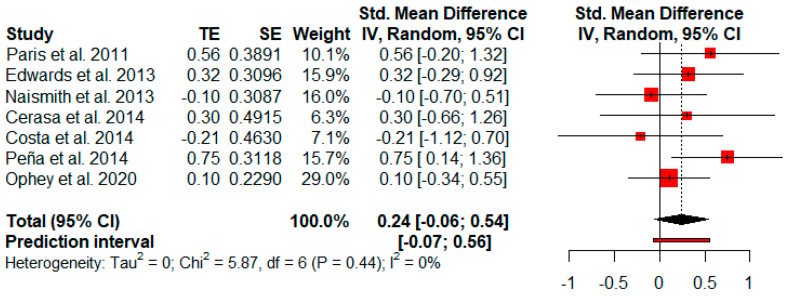
Effectiveness of cognitive rehabilitation in processing speed.

Seven studies reported processing speed outcomes. The random-effects model showed a small and not significant effect size (*g* = 0.24, *p* = 0.09) with a 95% CI (−0.06 to 0.54). The heterogeneity test showed low levels of heterogeneity across the studies (Q = 5.87, *p* = 0.44; *I*^2^ = 0%) (Figure 11).

#### 3.3.11. Others: Functionality, Depressive Symptoms and Quality of Life

**Figure 12:**
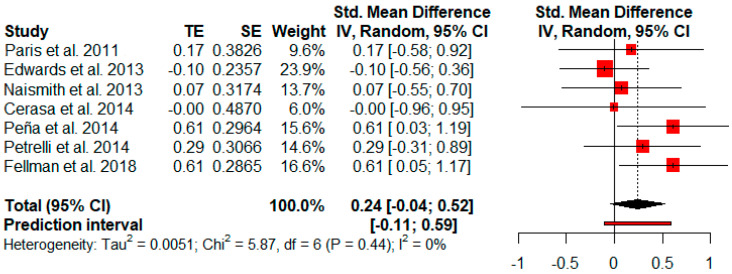
Effectiveness of cognitive rehabilitation in depressive symptoms.

Functionality was only reported in two studies, and so a meta-analysis could not be performed. Seven studies evaluated depressive symptoms after cognitive rehabilitation. Depressive symptoms showed a small and not significant effect size (*g* = 0.24, *p* = 0.08) with a 95% CI (−0.04 to 0.52). The heterogeneity test showed low levels of heterogeneity (Q = 5.87, *p* = 0.44; *I*^2^ = 0%) (Figure 12).

**Figure 13:**
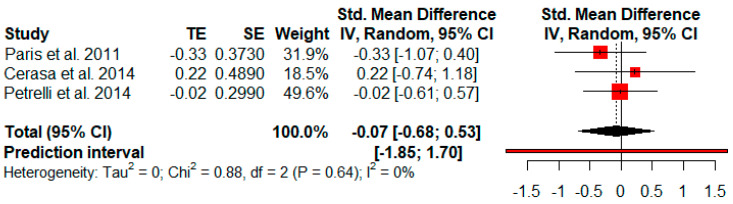
Effectiveness of cognitive rehabilitation in quality of life.

Only three studies reported quality of life outcomes. The random-effects model showed no effect size (*g* = −0.07, *p* = 0.64) with a broad 95% CI (−0.68 to 0.53). The heterogeneity test showed low levels of heterogeneity across the studies (Q = 0.88, *p* = 0.64; *I*^2^ = 0%) (Figure 13).

### 3.4. Moderator Analyses

Moderator analyses were performed to explore possible parameters that may explain the differences between the effect sizes obtained. The mixed-effects model meta-regression analyses were conducted with the overall cognitive function effect sizes estimated as previously shown in Figure 2, and with the following predictor variables: participants’ age and years of education, variables related to intervention time (number, frequency, and duration of sessions), the modality of delivering the cognitive training (pencil and paper or computer), baseline global cognitive scores and PD patient-related features (disease duration, UPDRS III [26] and H&Y scale [27]) (See Table 2).

No significant effects were found among the main effects of the predictor variables (models 1 to 10). We also tested the interaction between predictor variables (models 11 to 16) through a meta-regression analysis. Models 11, 12, 13, 14 and 16 showed no significant interactions. In contrast, the interaction between the number of sessions conducted and frequency of weekly sessions (model 15) was statistically significant and negatively associated with the overall cognitive function effect sizes (*F* (3, 5) = 4.4; β = −0.02; *p* = 0.04). These results indicated that the number of sessions in a cognitive rehabilitation programme could influence the effect sizes obtained when the weekly frequency of these sessions is also taken into account. Nonetheless, this interaction did not explain the possible heterogeneity between the different effect sizes (*R^2^* = 0%).

### 3.5. Publication Bias

Funnel plots and Eggers regression test were performed to analyze the presence of publication bias.

The funnel plot of overall cognitive functions, global cognitive status, cognitive subdomains, depressive symptoms and quality of life showed no evidence of asymmetry (see Figure S1). In addition, the results obtained from Egger’s regression test were not significant for most of the variables (*p* > 0.15), although a significant result was found in the attention domain (*p* = 0.01). However, Egger’s test does not provide sufficient information for global cognitive status, cognitive subdomains, depressive symptoms and quality of life, as there are fewer than 10 studies in each of the domains [48]. Additionally, although most of the results indicate low levels of publication bias, it is necessary to consider the levels in isolation and to consider the presence of certain levels of publication bias.

### 3.6. Sensitivity Analyses

Sensitivity analyses were conducted to examine whether the previously obtained results would change if Naismith and colleagues’ non-randomized study [39] was excluded from the analyses. Therefore, the analyses were carried out on the verbal memory, verbal fluency, executive function and processing speed cognitive subdomains. The sensitivity analyses showed small changes in the effect sizes of verbal memory (from *g* = 0.41, *p* = 0.00; to *g* = 0.37, *p* = 0.01), verbal fluency (from *g* = 0.27, *p* = 0.11; to *g* = 0.32, *p* = 0.11) and executive function (from *g* = 0.30, *p* = 0.04; to *g* = 0.30, *p* = 0.03) domains. Despite this, the exclusion of the non-randomized study did not significantly change the size nor the significance of the previous domains. The exception was observed in the processing speed cognitive domain, which, regardless of continuing to have a small effect size (from *g* = 0.24 to *g* = 0.31), becomes marginally significant (from *p* = 0.09 to *p* = 0.06) (see Figure S2).

## 4. Discussion

The aim of this study was to conduct a systematic review and meta-analysis of the literature regarding the effectiveness of cognitive rehabilitation in PD, not only at a cognitive level but also in functionality, depressive symptomatology and quality of life.

The systematic review indicated that the cognitive domains that most frequently improved after cognitive rehabilitation in PD were attention, working memory, verbal memory, executive functions and processing speed. Despite the considerable diversity and variability in intervention strategies, studies revealed cognitive improvements in at least one cognitive domain. Thus, cognitive rehabilitation has a positive impact on the cognition of PD patients, regardless of intervention method, duration and frequency [11].

The main results obtained from the meta-analysis showed moderate improvements in global cognitive status (*g* = 0.55) and working memory (*g* = 0.50). Small but significant improvements were found in verbal memory (*g* = 0.41), overall cognitive functions (*g* = 0.39) and executive functions (*g* = 0.30). Attention (*g* = 0.36), visual memory (*g* = 0.29), verbal fluency (*g* = 0.27) and processing speed (*g* = 0.24) showed a small non-significant effect and visuospatial and visuoconstructive abilities (*g* = 0.17) showed no effect. However, it should be noted that small effect sizes also report improvements after cognitive rehabilitation.

It is important to highlight that there was a moderate effect on global cognitive status after cognitive rehabilitation, and so it could be interesting to include global screening tests as outcome measures in the rehabilitation studies and not only to report them at baseline, in order to evaluate the differences between groups. On the other hand, the small effect size of overall cognitive functions showed in this meta-analysis may be due to the data analysis criteria applied in the article, which was based on the average of the global cognitive status and the cognitive subdomains of studies that had reported at least two cognitive variables.

Memory was one of the most frequently trained domains during the cognitive rehabilitation (5 of 12 studies), and was usually trained with a general approach, rather than being divided into verbal and visual memory domains. However, working memory was trained independently in some of the studies (four studies) and the results obtained from our meta-analysis showed a moderate and significant effect in this cognitive subdomain. In the case of the verbal memory and visual memory domains, 7 of the 12 studies included reported verbal memory measures and five reported visual memory. However, only four studies (two for verbal memory and two for visual memory) found significant improvements after rehabilitation. In this meta-analysis, we analyzed the effect sizes of verbal and visual memory independently, obtaining a small and significant effect size for verbal memory, although a small but non-significant effect size was found for visual memory. A meta-analysis performed in PD patients with MCI or dementia also obtained small effect sizes in verbal and visual memory [19]. Other meta-analyses analyzed improvements in memory using a general memory domain in which they also found a small effect after cognitive rehabilitation [17,18]. Regarding executive functions and processing speed, both domains were also two of the most frequently trained functions in cognitive rehabilitation (6–7/12 studies). The results obtained from our meta-analysis showed a small and significant effect size on executive functions, while processing speed showed a small and non-significant effect size. However, the small and significant effect size obtained in executive functions, and the small and not significant effect size in processing speed, may be due to the fact that executive functions and processing speed were two of the domains with the greatest variability among the assessment measures used. The effect sizes obtained in executive functions were similar to the meta-analysis performed in PD patients with MCI or dementia [19]. However, the meta-analyses conducted, respectively, by Leung and Lawrence [17,18] obtained moderate effect sizes in executive functions and small sizes in processing speed. This is the first meta-analysis of cognitive rehabilitation in PD that has analyzed the effects of verbal fluency after intervention and our results showed a small effect size in this domain. These results may have been obtained because although six studies reported verbal fluency measures, only one study showed improvements, specifically in semantic fluency.

Furthermore, changes in functionality, depressive symptoms and quality of life are also reported via transference effects [14], even though these functions have not been directly trained. A systematic review showed that one study has reflected the benefits of cognitive rehabilitation on the depressive symptomatology of people with PD. The small effect size (*g* = 0.24) obtained in the meta-analysis supports the results obtained from the systematic review, in which most of the studies that assessed depressive symptoms reported that the patients with PD obtained similar scores to those obtained at the beginning of the cognitive intervention. Regarding quality of life, no evidence was found after cognitive training. Therefore, further studies that include quality of life, functionality and mood as outcome variables need to be conducted.

There were low levels of heterogeneity and publication bias in most of the outcomes analyzed. Besides, we carried out moderator analyses only for the overall cognitive functions, and the results suggest that the interaction between the number of sessions conducted and the frequency of sessions in a cognitive training program could be relevant variables to take into account when applying and designing a cognitive rehabilitation program. However, none of them could explain the differences between the effect sizes obtained.

There are several limitations in this systematic review and meta-analysis study. First, the small number of studies that met the inclusion criteria for this meta-analysis limited the precision of the publication bias. In addition, the lack of data or the use of different methods in the analyses limits the possibility of making a comparison between all the studies included in the systematic review. On the one hand, the variability of trained cognitive functions allows studies divided by different cognitive domains to be compared, although not all the cognitive domains were trained in all the studies and so, in some cases, the comparison would be limited. On the other hand, some of the studies measured the cognitive domains differently. Therefore, when the specific cognitive domains were grouped according to the tests used, only studies that reported those tests independently could be included.

## 5. Importance of Cognitive Rehabilitation in Personalized Medicine

Personalized medicine refers to the idea that treatment strategies could be influenced by age, personality, lifestyle, genetic factors, pharmacoeconomics, pharmacogenetics and comorbidity [10,49]. Personalized medicine seeks to consider these aspects in order to develop individualized treatment strategies for each person with PD [10,49]. In this line, cognitive rehabilitation is proposed as an option in personalized medicine strategy to manage cognitive impairment [10,49], and it can be administered, along with other pharmacological therapies, to improve cognitive impairment [50].

This systematic review and meta-analysis showed the effectiveness of cognitive rehabilitation in PD. It is important to analyze the variables that influence the cognitive rehabilitation process itself in order to elucidate which are the best components of a successful cognitive rehabilitation program. Some of these variables are: age, years of education, the variables related to the time of intervention (number, frequency and duration of sessions), the modality of the cognitive training (pencil and paper or computer), the baseline global cognitive scores and PD patient-related features (disease duration, UPDRS and H&Y stages). However, in the regression analysis of our meta-analysis, none of these variables showed a percentage of variance accounted for in the effect sizes. Studies should continue reporting the specific methodology used and provide as much detailed information as possible to further investigate these aspects. Regarding age, studies in neuropsychiatric diseases such as schizophrenia showed that younger participants obtained a greater benefit from cognitive rehabilitation [51,52]. In PD studies, the groups that perform cognitive rehabilitation activities usually have a similar average age, which allows for more accurate comparison between groups. However, further studies are needed that analyze the age factor, because the age at the time of the diagnosis, the cognitive impairment associated with age and the age at the time of the intervention are all variables that could influence the cognitive rehabilitation process. In addition, it is important to start cognitive rehabilitation as early as possible in order to manage the cognitive deficits in PD patients. Most studies focus on participants at the early stages of the disease (H&Y stages ≤3), since the application of treatment before the onset of dementia could increase or maintain cognitive outcomes over time [12]. However, few studies focus on analyzing the impact of cognitive rehabilitation in more advanced stages of the disease, including patients with PD dementia. Previous meta-analyses [17,18], as well as our meta-analysis, focused on analyzing the impact of cognitive rehabilitation in PD without dementia and found evidence of cognitive improvement. In PD patients with dementia, no evidence of improvement has been found after cognitive rehabilitation [19]. Therefore, it is very important to start the cognitive intervention before the appearance of cognitive deterioration. However, it would be interesting to continue investigating the effects of cognitive rehabilitation in PD-MCI and dementia, and to observe whether, despite the presence of dementia, it is possible to maintain or reduce the progression of cognitive deficits.

Active lifestyle and personality are two determining factors in the cognitive rehabilitation process. Participating in a specific cognitive rehabilitation program requires the availability of sufficient time for attending sessions and performing cognitive activities at set times. A proactive, active lifestyle and a positive attitude can help in increasing attendance and participation in cognitive rehabilitation sessions and achieving positive results. A study conducted in older adults at risk of dementia analyzed the relationship between self-reported lifestyle and cognitive changes associated with cognitive and physical rehabilitation [53]. The authors showed that individuals with a more active lifestyle demonstrated a favorable change in cognitive performance during the study period compared to individuals with a less active lifestyle, regardless of the group to which they belonged (experimental or control groups) [53]. In addition, the personality of each individual may also influence participation in their rehabilitation program, as well as how comfortable they feel with therapy (group or individual). A common component regarding PD patients’ personal factors is whether intervention groups should be homogeneous or heterogeneous. Homogeneous groups can facilitate the integration of each participant more easily because all members experience similar conditions. However, it is sometimes necessary to consider that heterogeneous groups can encourage companionship and prompt people to help each other.

Finally, another important variable to consider in personalized medicine is genetics. In our meta-analysis, the included studies were focused on idiopathic PD, without mentioning genetic variants of PD. However, PD is a heterogeneous disease in which 3–5% of cases are affected by a genetic variant [54], which contributes to clinical variability, in some cases also including a predisposition to cognitive impairment and dementia [55]. Therefore, further studies should be conducted to analyze the effect of cognitive rehabilitation in genetic PD, especially in patients with a mutation known to cause a higher predisposition to cognitive impairment.

## 6. Conclusions

The review of available studies along with the effect sizes obtained in the meta-analysis would seem to support the fact that cognitive rehabilitation may be beneficial in improving cognitive functions in PD patients. However, there are not many studies that assess functionality and quality of life after cognitive rehabilitation. Therefore, more studies are needed to analyze the effectiveness of cognitive rehabilitation in patients with PD at the cognitive level and on the instrumental activities of daily living, functionality and quality of life. In addition, it would be interesting to analyze individual factors such as age, lifestyle, personality and genetic factors, which may be applicable to personalized medicine, in order to design more specific and individualized interventions. On the other hand, cognitive impairment, dysfunctionality and disease progression in people with PD are determining factors in the quality of life of patients and their family caregivers, resulting in major changes in their lives and creating a future need for long-term care. This care is usually provided by a family member [56], leading to possible physical, emotional and psychosocial problems for the caregivers themselves [57]. It is necessary to include treatment that takes a holistic approach to the disease, and thus, incorporating systems of psychoeducation and measurement of clinical symptoms may be beneficial for people with PD and their family caregivers.

## Figures and Tables

**Table 1:** Summary of cognitive rehabilitation studies for PD.

Authors	Sample	Characteristics of the Sample	H&Y Stages	Intervention	Format	Duration	Domains Trained	Outcomes
Sammer et al., 2006 [36]	26 PD 12 PD-CR 14 PD-ACG	Age: 70.8 PD-CR 68.5 PD-ACG Years of education: -	2–3	CR: Executive Function Training (Paper) ACG: Standard Treatment (Occupational Therapy, Physical Therapy and Treatment) (Paper)	Group	10 sessions 30 min/session	-Working memory -Executive functions	Improvements in PD-CR: -Executive functions
París et al., 2011 [37]	28 PD 12 PD-CR 16 PD-CG	Age: 64.7 PD-CR 65.4 PD-CG Years of education: 9.8 PD-CR 9.5 PD-CG	1–3	“SmartBrain Tool” (Paper + Computer)	Group + home	12 sessions 3 times/week 4 weeks 45 min/session	-Attention -Memory -Psychomotor speed -Executive functions -Visuospatial abilities -Language -Calculation abilities	Improvements in PD-CR: -Attention -Processing speed -Visual memory -Visuospatial and visuoconstructive abilities -Semantic fluency -Executive functions
Edwards et al., 2013 [38]	74 PD 32 PD-CR 42 PD-CG	Age: 69.4 PD-CR 68.2 PD-CG Years of education: 14.8 PD-CR 15.4 PD-CG	1–3	“Insight Software” (Computer)	Home	36 sessions 3 times/week 3 months 60 min/session	-Processing speed	Improvements in PD-CR: -Processing speed
Naismith et al., 2013 [39]	50 PD 35 PD-CR 15 PD-CG	Age: 68.5 PD-CR 64.9 PD-CG Years of education: 14.9 PD-CR 14.0 PD-CG	1–3	Cognitive training (Neuropsychological Educational Approach to Remediation (NEAR)) + Psychoeducation (Computer)	Group	14 sessions 7 weeks 2 times/week 60 min of CR 60 min psycho-education	-Memory -Psychomotor speed -Mental flexibility -Verbal fluency	Improvements in PD-CR: -Verbal memory
Cerasa et al., 2014 [40]	15 PD 8 PD-CR 7 PD-CG	Age: 61.1 PD-CR 58.3 PD-CG Years of education: 8 PD-CR 8 PD-CG	1–3	CR: “Rehacom” CG: Simple visuomotor coordination tapping task (Computer)	Group (CR) + Individual (CG)	6 weeks 2 times/week 60 min/session	-Attention -Information processing task	Improvements in PD-CR: -Attention/ Processing speed -Working memory Increased functional brain activity: -Left dorsolateral prefrontal cortex -Superior left parietal cortex
Costa et al., 2014 [41]	17 PD-MCI 9 PD-CR 8 PD-CG	Age: 66.1 PD-CR 70.9 PD-CG Years of education: 11.2 PD-CR 10.6 PD-CG	1–3	CR: Cognitive change training CG: Breathing training (Paper)	-	4 weeks 3 times/week 45 min/session	-Shifting abilities (Verbal fluency and Trail Making Test)	Improvements in PD-CR: -Verbal fluency -Trail Making Test -Prospective memory procedures
Peña et al., 2014 [42]	42 PD 20 PD-CR 22 PD-ACG	Age: 67.7 PD-CR 68.1 PD-ACG Years of education: 10.5 PD-CR 10.2 PD-ACG	1–3	CR: “REHACOP” ACG: Occupational activities (Paper)	Group	39 sessions 13 weeks 2 times/week 60 min/session	-Attention -Memory -Language -Executive functions -Social cognition -Processing speed	Improvements in PD-CR: -Processing speed -Visual memory -Social cognition -Functional disability
Petrelli et al., 2014 [43]	65 PD 22 PD-NV 22 PD-MF 21 PD-CG	Age: 69.2 PD-NV 68.8 PD-MF 69.1 PD-CG Years of education: 13.1 PD-NV 13.6 PD-MF 12.8 PD-CG	1–3	NV: “NEUROvitalis” (Computer) MF: “Mentally fit” (Paper)	Group + Individual	12 sessions 6 weeks 2 times/week 90 min/session	NV -Attention -Verbal memory -Visual memory -Executive functions MF -Attention -Memory -Creativity	Improvements in PD-NV versus PD-CG: -Working memory -Verbal memory Improvements in PD-MF versus PD-CG: -Depressive symptoms Improvements in PD-NV versus PD-MF: -Working memory
Angelucci et al., 2015 [44]	15 PD-MCI 7 PD-CR 8 PD-CG	Age: 67.6 PD-CR 71.9 PD-CG Years of education: 11.7 PD-CR 10.6 PD-CG	-	CR: Cognitive change training CG: Simple cognitive tests (Paper)	-	12 sessions 4 weeks 3 times/week 45 min/session	-Executive functions	Improvements in PD-CR: -Executive functions
Fellman et al., 2018 [45]	52 PD 26 PD-CR 26 PD-ACG	Age: 64.8 PD-CR 65.5 PD-ACG Years of education: 5.3 PD-CR 5.5 PD-ACG	-	CR: Working memory ACG: Online quiz task “Älypää” (Computer)	Home	3 weeks 3 times/week 30 min/session	-Working memory	Improvements in PD-CR: -Working memory -Depressive symptoms
Bernini et al., 2020 [46]	53 PD 18 PD-CCT 12 PD-PCT 18 PD-CG	Age: 74.6 PD-CCT 69.8 PD-PCT 69.3 PD-CG Years of education: 9.5 PD-CCT 8.0 PD-PCT 7.6 PD-CG	≤3	CCT: “CoRe” (Computer) PCT: “CoRe” (Paper) CG: Unstructured activities	Group	3 weeks 4 times/week 45 min/session	-Logical executive functions -Attention/processing speed -Working memory -Episodic long-term memory	Improvements in PD-CCT versus PD-PCT: -Global cognition Improvements in PD-CCT versus CG: -Global cognition -Attention/processing speed
Ophey et al., 2020 [47]	75 PD 37 PD-CR 38 PD-CG	Age: 64.0 PD-CR 63.8 PD-CG Years of education: 15.0 PD-CR 15.5 PD-CG	2–3	CR: “NeuroNation” (Computer)	Individual + home	5 weeks 5 times/week 30 min/session	-Working memory	Improvements in PD-CR: -No improvements Assesment after 3 months: -Verbal working memory -Visuoconstructive abilities

PD = Parkinson’s disease; CR = cognitive rehabilitation; ACG = active control group; CCT = computer cognitive training; PCT = paper-pencil cognitive training; CG = control group; H&Y = Hoehn and Yahr scale; MCI = Mild cognitive impairment

**Table 2:** Results of overall cognitive function meta-regression analyses.

Model Number	Predictor Variables	k	df	*F*_moderator_	Q_residual_	*R* ^2^	β	*p*
1	Age of participants	10	8	3.96	2.70	0%	0.04	0.08
2	Years of education	9	7	4.45	4.45	0%	−0.03	0.07
3	H&Y	8	6	2.03	2.79	0%	0.66	0.20
4	Duration of PD (years)	9	7	1.10	2.36	0%	0.01	0.32
5	Baseline global cognitive scores	10	8	0.30	3.90	0%	0.00	0.60
6	Total number of conducted sessions	10	8	0.02	4.03	0%	−0.00	0.89
7	Training session duration (min)	10	8	0.02	4.03	0%	0.01	0.87
8	Frequency of weekly sessions	9	7	1.72	2.19	0%	−0.08	0.23
9	UPDRS-III	8	6	0.03	2.4	0%	0.01	0.87
10	Tools for cognitive training (pencil & paper or computer)	10	7	1.71	2.71	0%	−0.12	0.24
11	Age × Years of education	9	5	2.71	1.04	0%	0.01	0.15
12	Duration of PD × H&Y	8	4	1.70	1.64	0%	−0.06	0.30
13	H&Y × UPDRS-III	6	2	0.43	1.05	0%	−1.54	0.75
14	Total number of sessions conducted × Training session duration (min)	10	6	4.04	1.33	0%	0.00	0.07
15	Total number of sessions conducted × Frequency of weekly sessions	9	5	4.41	0.75	0%	−0.02	0.04 *
16	Training session duration (min) × Frequency of weekly sessions	9	5	2.21	1.17	0%	0.01	0.20

Note: k *=* number of studies; *F*_moderator_ *=* test of moderators; Q_residual_ *=* test for residual heterogeneity; *R^2^ =* amount of heterogeneity accounted for; β *=* estimate; * *= p* < 0.05; PD *=* Parkinson’s disease; H&Y *=* Hoehn and Yahr scale; UPDRS-III *=* Unified Parkinson’s Disease Rating Scale-part III.

## Data Availability

All relevant data are included in the study and supplementary information.

## Supplementary Materials

The following are available online at https://www.mdpi.com/article/10.3390/jpm11050429/s1, Figure S1: Funnel plot asymmetry of publication bias, Figure S2: Forest plot of sensitive analysis: Effect sizes of randomized controlled trials, Table S1: Search strategy, Table S2: Methodological quality and risk of bias, Table S3: Outcomes and assessment included in the meta-analysis.

## References

[1] Poewe W., Seppi K., Tanner C.M., Halliday G.M., Brundin P., Volkmann J., Schrag A.-E., Lang A.E. (2017). Parkinson disease. Nat. Rev. Dis. Prim..

[2] Schapira A.H., Chaudhuri K.R., Jenner P. (2017). Non-motor features of Parkinson disease. Nat. Rev. Neurosci..

[3] Emre M., Aarsland D., Brown R., Burn D.J., Duyckaerts C., Mizuno Y., Broe G.A., Cummings J., Dickson D.W., Gauthier S. (2007). Clinical diagnostic criteria for dementia associated with Parkinson’s disease. Mov. Disord..

[4] Baiano C., Barone P., Trojano L., Santangelo G. (2020). Prevalence and clinical aspects of mild cognitive impairment in Parkinson’s disease: A meta-analysis. Mov. Disord..

[5] Svenningsson P., Westman E., Ballard C., Aarsland D. (2012). Cognitive impairment in patients with Parkinson’s disease: Diagnosis, biomarkers, and treatment. Lancet Neurol..

[6] Aarsland D., Creese B., Politis M., Chaudhuri K.R., Ffytche D.H., Weintraub D., Ballard C. (2017). Cognitive decline in Parkinson disease. Nat. Rev. Neurol..

[7] Sasikumar S., Strafella A.P. (2020). Imaging mild cognitive impairment and dementia in Parkinson’s disease. Front. Neurol..

[8] Hely M.A., Reid W.G., Adena M.A., Halliday G.M., Morris J.G. (2008). The Sydney multicenter study of Parkinson’s disease: The inevitability of dementia at 20 years. Mov. Disord..

[9] Van de Weijer S., Hommel A., Bloem B., Nonnekes J., De Vries N. (2018). Promising non-pharmacological therapies in PD: Targeting late stage disease and the role of computer based cognitive training. Park. Relat. Disord..

[10] Titova N., Chaudhuri K.R. (2017). Personalized medicine in Parkinson’s disease: Time to be precise. Mov. Disord..

[11] Alzahrani H., Venneri A. (2018). Cognitive rehabilitation in Parkinson’s disease: A systematic review. J. Park. Dis..

[12] Biundo R., Weis L., Fiorenzato E., Antonini A. (2017). Cognitive rehabilitation in Parkinson’s disease: Is it feasible?. Arch. Clin. Neuropsychol..

[13] Couture M., Giguère-Rancourt A., Simard M. (2018). The impact of cognitive interventions on cognitive symptoms in idiopathic Parkinson’s disease: A systematic review. Aging Neuropsychol. Cogn..

[14] Díez-Cirarda M., Ibarretxe-Bilbao N., Peña J., Ojeda N. (2018). Neurorehabilitation in Parkinson’s disease: A critical review of cognitive rehabilitation effects on cognition and brain. Neural Plast..

[15] Hindle J.V., Petrelli A., Clare L., Kalbe E. (2013). Nonpharmacological enhancement of cognitive function in Parkinson’s disease: A systematic review. Mov. Disord..

[16] Glizer D., MacDonald P.A. (2016). Cognitive training in Parkinson’s disease: A review of studies from 2000 to 2014. Park. Dis..

[17] Lawrence B.J., Gasson N., Bucks R.S., Troeung L., Loftus A.M. (2017). Cognitive training and noninvasive brain stimulation for cognition in Parkinson’s disease: A meta-analysis. Neurorehabilit. Neural Repair.

[18] Leung I.H., Walton C.C., Hallock H., Lewis S.J., Valenzuela M., Lampit A. (2015). Cognitive training in Parkinson disease. Neurology.

[19] Orgeta V., McDonald K.R., Poliakoff E., Hindle J.V., Clare L., Leroi I. (2020). Cognitive training interventions for dementia and mild cognitive impairment in Parkinson’s disease. Cochrane Database Syst. Rev..

[20] Vlagsma T.T., Koerts J., Fasotti L., Tucha O., Van Laar T., Dijkstra H., Spikman J.M. (2015). Parkinson’s patients’ executive profile and goals they set for improvement: Why is cognitive rehabilitation not common practice?. Neuropsychol. Rehabil..

[21] Page M.J., Moher D., Bossuyt P.M., Boutron I., Hoffmann T.C., Mulrow C.D., Shamseer L., Tetzlaff J.M., Akl E., Brennan S. (2021). PRISMA 2020 explanation and elaboration: Updated guidance and exemplars for reporting systematic reviews. BMJ.

[22] Sterne J.A.C., Savović J., Page M.J., Elbers R.G., Blencowe N.S., Boutron I., Cates C.J., Cheng H.-Y., Corbett M.S., Eldridge S.M. (2019). RoB 2: A revised tool for assessing risk of bias in randomised trials. BMJ.

[23] Sterne J.A., Hernán M.A., Reeves B.C., Savović J., Berkman N.D., Viswanathan M., Henry D., Altman D.G., Ansari M.T., Boutron I. (2016). ROBINS-I: A tool for assessing risk of bias in non-randomised studies of interventions. BMJ.

[24] Higgins J.P.T., Savović J., Page M.J., Elbers R.G., Sterne J.A.C., Higgins J.P.T., Thomas J., Chandler J., Cumpston M., Li T., Page M.J., Welch V.A. (2021). Chapter 8: Assessing risk of bias in a randomized trial. Cochrane Handbook for Systematic Reviews of Interventions.

[25] Maher C.G., Sherrington C., Herbert R.D., Moseley A.M., Elkins M. (2003). Reliability of the PEDro scale for rating quality of randomized controlled trials. Phys. Ther..

[26] Martínez-Martín P., Gil-Nagel A., Gracia L.M., Gómez J.B., Martínez-Sarriés J., Bermejo F. (1994). The Cooperative Multicentric Group Unified Parkinson’s disease rating scale characteristics and structure. Mov. Disord..

[27] Hoehn M.M., Yahr M.D. (1967). Parkinsonism: Onset, progression, and mortality. Neurology.

[28] Cohen J. (1988). Statistical Power Analysis for the Behavioral Sciences.

[29] Cumming G. (2012). Understanding the New Statistics: Effect Sizes, Confidence Intervals, and Meta-Analysis.

[30] Huedo-Medina T.B., Sánchez-Meca J., Marín-Martínez F., Botella J. (2006). Assessing heterogeneity in meta-analysis: Q statistic or I² index?. Psychol. Methods.

[31] Harrer M., Cuijpers P., Furukawa T., Ebert D.D. (2019). Doing Meta-Analysis in R: A Hands-on Guide.

[32] Cheung M.W.-L., Vijayakumar R. (2016). A Guide to Conducting a Meta-Analysis. Neuropsychol. Rev..

[33] Dickersin K. (1990). The existence of publication bias and risk factors for its occurrence. JAMA.

[34] Rothstein H.R., Sutton A.J., Borenstein M. (2006). Publication Bias in Meta-Analysis.

[35] Egger M.C.M., Smith G.D., Schneider M., Minder C. (1997). Bias in meta-analysis detected by a simple, graphical test. Br. Med. J..

[36] Sammer G., Reuter I., Hullmann K., Kaps M., Vaitl D. (2006). Training of executive functions in Parkinson’s disease. J. Neurol. Sci..

[37] París A.P., Saleta H.G., Maraver M.D.L.C.C., Silvestre E., Freixa M.G., Torrellas C.P., Pont S.A., Nadal M.F., Garcia S.A., Bartolomé M.V.P. (2011). Blind randomized controlled study of the efficacy of cognitive training in Parkinson’s disease. Mov. Disord..

[38] Edwards J.D., Hauser R.A., O’Connor M.L., Valdés E.G., Zesiewicz T.A., Uc E.Y. (2013). Randomized trial of cognitive speed of processing training in Parkinson disease. Neurology.

[39] Naismith S.L., Mowszowski L., Diamond K., Lewis S.J. (2013). Improving memory in Parkinson’s disease: A healthy brain ageing cognitive training program. Mov. Disord..

[40] Cerasa A., Gioia M.C., Salsone M., Donzuso G., Chiriaco C., Realmuto S., Nicoletti A., Bellavia G., Banco A., D’Amelio M. (2014). Neurofunctional correlates of attention rehabilitation in Parkinson’s disease: An explorative study. Neurol. Sci..

[41] Costa A., Peppe A., Serafini F., Zabberoni S., Barban F., Caltagirone C., Carlesimo G.A. (2014). Prospective memory performance of patients with Parkinson’s disease depends on shifting aptitude: Evidence from cognitive rehabilitation. J. Int. Neuropsychol. Soc..

[42] Peña J., Ibarretxe-Bilbao N., García-Gorostiaga I., Gomez-Beldarrain M.A., Díez-Cirarda M., Ojeda N. (2014). Improving functional disability and cognition in Parkinson disease: Randomized controlled trial. Neurology.

[43] Petrelli A., Kaesberg S., Barbe M.T., Timmermann L., Fink G.R., Kessler J., Kalbe E. (2014). Effects of cognitive training in Parkinson’s disease: A randomized controlled trial. Park. Relat. Disord..

[44] Eangelucci F., Epeppe A., Carlesimo G.A., Eserafini F., Ezabberoni S., Ebarban F., Eshofany J., Ecaltagirone C., Ecosta A. (2015). A pilot study on the effect of cognitive training on BDNF serum levels in individuals with Parkinson’s disease. Front. Hum. Neurosci..

[45] Fellman D., Salmi J., Ritakallio L., Ellfolk U., Rinne J.O., Laine M. (2018). Training working memory updating in Parkinson’s disease: A randomised controlled trial. Neuropsychol. Rehabil..

[46] Bernini S., Panzarasa S., Barbieri M., Sinforiani E., Quaglini S., Tassorelli C., Bottiroli S. (2020). A double-blind randomized controlled trial of the efficacy of cognitive training delivered using two different methods in mild cognitive impairment in Parkinson’s disease: Preliminary report of benefits associated with the use of a computerized tool. Aging Clin. Exp. Res..

[47] Ophey A., Giehl K., Rehberg S., Eggers C., Reker P., van Eimeren T., Kalbe E. (2020). Effects of working memory training in patients with Parkinson’s disease without cognitive impairment: A randomized controlled trial. Park. Relat. Disord..

[48] Sterne J.A.C., Sutton A.J., Ioannidis J.P.A., Terrin N., Jones D.R., Lau J., Carpenter J., Rücker G., Harbord R.M., Schmid C.H. (2011). Recommendations for examining and interpreting funnel plot asymmetry in meta-analyses of randomised controlled trials. BMJ.

[49] Titova N., Chaudhuri K.R. (2017). Personalized medicine and nonmotor symptoms in Parkinson’s diseas. Int. Rev. Neurobiol..

[50] Calleo J., Burrows C., Levin H., Marsh L., Lai E., York M.K. (2011). Cognitive rehabilitation for executive dysfunction in Parkinson’s disease: Application and current directions. Park. Dis..

[51] Wykes T., Reeder C., Landau S., Matthiasson P., Haworth E., Hutchinson C. (2009). Does age matter? Effects of cognitive rehabilitation across the age span. Schizophr. Res..

[52] Kontis D., Huddy V., Reeder C., Landau S., Wykes T. (2013). Effects of age and cognitive reserve on cognitive remediation therapy outcome in patients with schizophrenia. Am. J. Geriatr. Psychiatry.

[53] Küster O.C., Fissler P., Laptinskaya D., Thurm F., Scharpf A., Woll A., Kolassa S., Kramer A.F., Elbert T., Von Arnim C.A.F. (2016). Cognitive change is more positively associated with an active lifestyle than with training interventions in older adults at risk of dementia: A controlled interventional clinical trial. BMC Psychiatry.

[54] Klein C., Westenberger A. (2012). Genetics of Parkinson’s disease. Cold Spring Harb. Perspect. Med..

[55] Fagan E.S., Pihlstrøm L. (2017). Genetic risk factors for cognitive decline in Parkinson’s disease: A review of the literature. Eur. J. Neurol..

[56] Peters M., Fitzpatrick R., Doll H., Playford D., Jenkinson C. (2011). Does self-reported well-being of patients with Parkinson’s disease influence caregiver strain and quality of life?. Park. Relat. Disord..

[57] Martínez-Martín P., Rodríguez-Blázquez C., Forjaz M.J. (2012). Quality of life and burden in caregivers for patients with Parkinson’s disease: Concepts, assessment and related factors. Expert Rev. Pharm. Outcomes Res..

