# Supplementary material for: Effectiveness of Cognitive Rehabilitation in Parkinson’s Disease: A Systematic Review and Meta-Analysis
Source: J Pers Med. 2021 May 18;11(5):429. doi: 10.3390/jpm11050429 (PMC8157874; doi:10.3390/jpm11050429)

Figure S2: Forest plot of sensitive analysis: Effect sizes of randomized controlled trials

Verbal memory

Study	TE	SE	Weight	Std. Mean Difference IV, Random, 95% CI
Paris et al. 2011	0.76	0.3950	10.1%	0.76 [-0.01; 1.53]
Cerasa et al. 2014	0.20	0.4890	6.6%	0.20 [-0.76; 1.16]
Peña et al. 2014	0.59	0.3079	16.7%	0.59 [-0.01; 1.19]
Petrelli et al. 2014	0.54	0.3106	16.4%	0.54 [-0.07; 1.15]
Fellman et al. 2018	0.25	0.2812	20.0%	0.25 [-0.30; 0.80]
Ophey et al. 2020	0.13	0.2290	30.2%	0.13 [-0.32; 0.58]
**Total (95% CI)**			**100.0%**	**0.37 [0.11; 0.62]**
**Prediction interval**				**[0.09; 0.65]**

Heterogeneity: $Tau^2 = 0$; $Chi^2 = 3.22$, df = 5 (P = 0.67); $I^2 = 0\%$

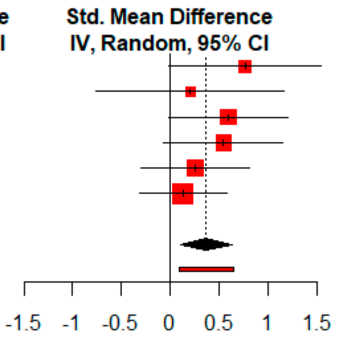

Verbal fluency

Study	TE	SE	Weight	Std. Mean Difference IV, Random, 95% CI
Paris et al. 2011	1.03	0.4061	13.8%	1.03 [0.24; 1.83]
Cerasa et al. 2014	0.20	0.4880	9.6%	0.20 [-0.76; 1.15]
Costa et al. 2014	0.57	0.4984	9.2%	0.57 [-0.41; 1.54]
Petrelli et al. 2014	0.31	0.3068	24.2%	0.31 [-0.29; 0.91]
Ophey et al. 2020	0.07	0.2300	43.1%	0.07 [-0.38; 0.52]
**Total (95% CI)**			**100.0%**	**0.32 [-0.13; 0.77]**
**Prediction interval**				**[-0.19; 0.83]**

Heterogeneity: $Tau^2 = 0$; $Chi^2 = 4.57$, df = 4 (P = 0.33); $I^2 = 12\%$

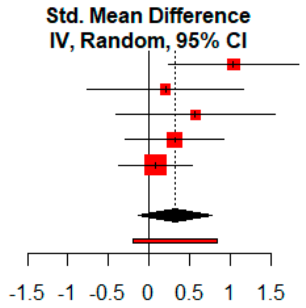

Executive functions

Study	TE	SE	Weight	Std. Mean Difference IV, Random, 95% CI
Sammer et al. 2006	0.73	0.4063	10.9%	0.73 [-0.07; 1.52]
Paris et el. 2011	0.60	0.3902	11.8%	0.60 [-0.16; 1.37]
Cerasa et al. 2014	-0.19	0.4740	8.0%	-0.19 [-1.12; 0.74]
Costa et al. 2014	0.57	0.4710	8.1%	0.57 [-0.35; 1.49]
Peña et al. 2014	0.31	0.3033	19.6%	0.31 [-0.28; 0.91]
Angelucci et al. 2015	0.54	0.4970	7.3%	0.54 [-0.43; 1.52]
Ophey et al. 2020	0.06	0.2290	34.3%	0.06 [-0.39; 0.51]
**Total (95% CI)**			**100.0%**	**0.30 [0.02; 0.59]**
**Prediction interval**				**[0.01; 0.60]**

Heterogeneity: $Tau^2 = 0$; $Chi^2 = 4.43$, df = 6 (P = 0.62); $I^2 = 0\%$

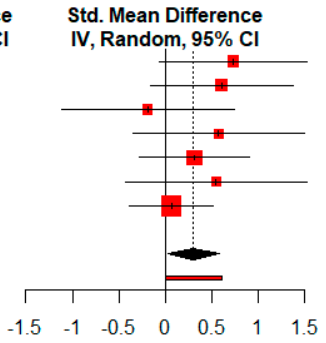

Processing speed

Study	TE	SE	Weight	Std. Mean Difference IV, Random, 95% CI
Paris et al. 2011	0.56	0.3891	12.0%	0.56 [-0.20; 1.32]
Edwards et al. 2013	0.32	0.3096	18.9%	0.32 [-0.29; 0.92]
Cerasa et al. 2014	0.30	0.4915	7.5%	0.30 [-0.66; 1.26]
Costa et al. 2014	-0.21	0.4630	8.5%	-0.21 [-1.12; 0.70]
Peña et al. 2014	0.75	0.3118	18.6%	0.75 [0.14; 1.36]
Ophey et al. 2020	0.10	0.2290	34.5%	0.10 [-0.34; 0.55]
**Total (95% CI)**			**100.0%**	**0.31 [-0.02; 0.63]**
**Prediction interval**				**[-0.05; 0.66]**

Heterogeneity: $Tau^2 = 0$; $Chi^2 = 4.44$, df = 5 (P = 0.49); $I^2 = 0\%$

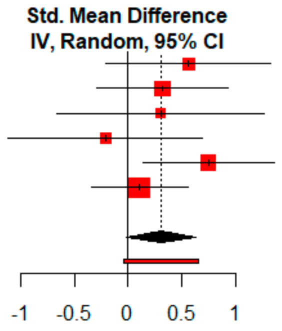

Supplement: Supplementary file 1 [file jpm-11-00429-s001.zip › SupplementaryMaterialFigure2_IbarretxeBilbao.pdf]
