# Supplementary material for: Effectiveness of Cognitive Rehabilitation in Parkinson’s Disease: A Systematic Review and Meta-Analysis
Source: J Pers Med. 2021 May 18;11(5):429. doi: 10.3390/jpm11050429 (PMC8157874; doi:10.3390/jpm11050429)

Supplementary material

Figure S1: Funnel plot asymmetry of publication bias

Overall cognition

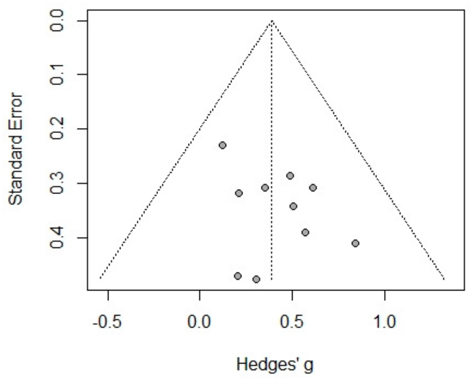

Global cognitive status

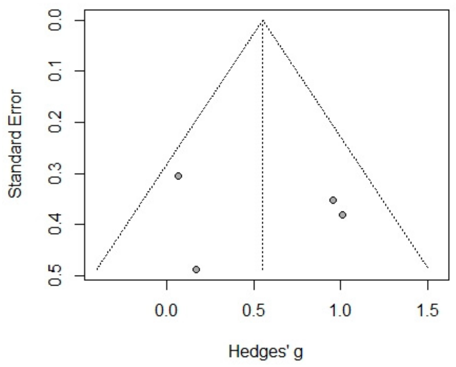

Attention

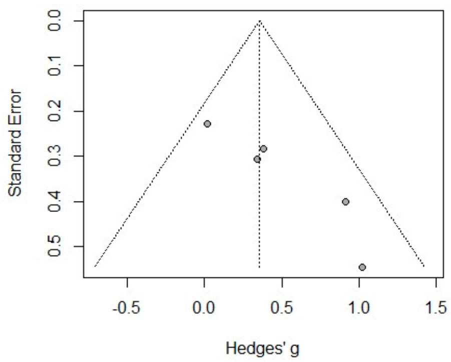

Working memory

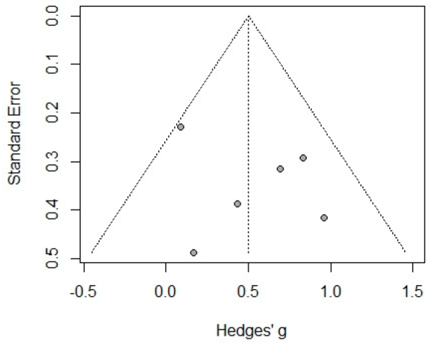

Verbal memory

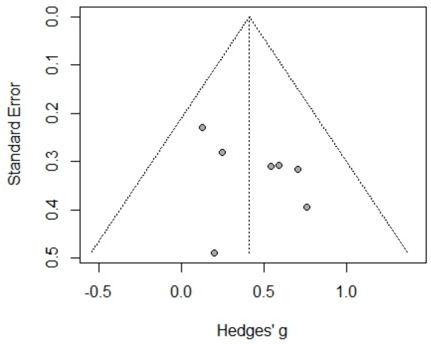

Visual memory

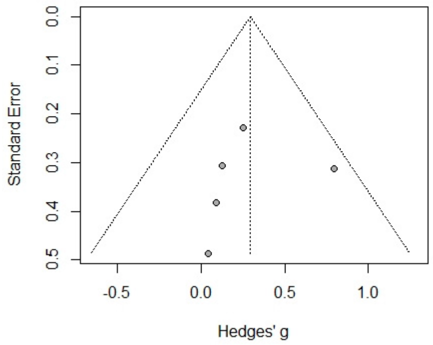

Verbal fluency

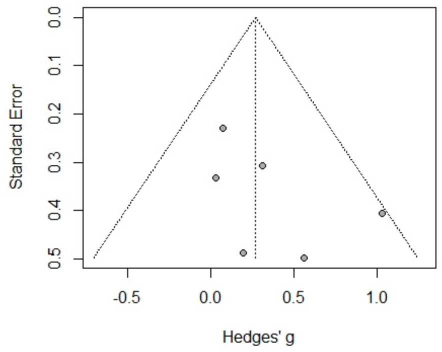

Executive functions

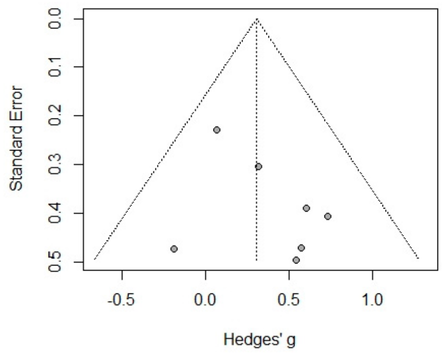

Visuospatial and visuoconstructive abilities

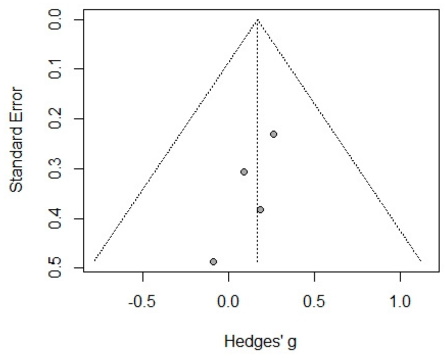

Processing speed

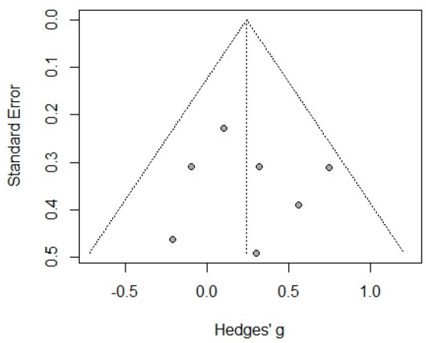

Depressive symptoms

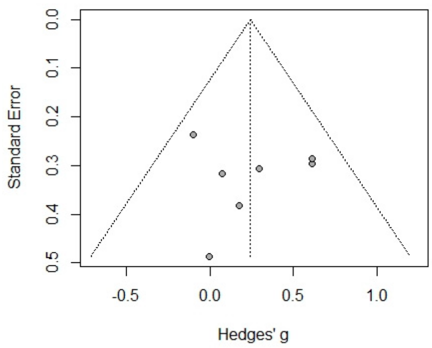

Quality of life

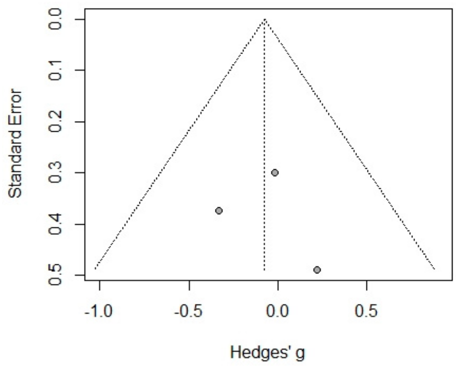

Supplement: Supplementary file 1 [file jpm-11-00429-s001.zip › SupplementaryMaterial_Figure1_IbarretxeBilbao.pdf]
