# Supplementary material for: Effectiveness of Cognitive Rehabilitation in Parkinson’s Disease: A Systematic Review and Meta-Analysis
Source: J Pers Med. 2021 May 18;11(5):429. doi: 10.3390/jpm11050429 (PMC8157874; doi:10.3390/jpm11050429)
Supplement: Supplementary file 1 [file jpm-11-00429-s001.zip › SupplementaryMaterial_Table1_SearchStrategy.pdf]

Supplementary material

Table S1: Search strategy

CONCEPT 1		CONCEPT 2		-	Number of obtained results (n= 1472)
(“Parkinson’s disease” OR “Parkinson disease”)	AND	("Cognitive rehabilitation" OR "Cognitive remediation" OR "Cognitive training" OR "Cognitive stimulation")		-	144
**CONCEPT 1**		**CONCEPT 2**		**CONCEPT 3**	-
(“Parkinson's disease” OR “Parkinson disease”)	**AND**	(Attention*)	**AND**	(Rehabilitation OR Remediation OR Training OR Stimulation)	527
(“Parkinson's disease” OR “Parkinson disease”)	**AND**	(“Working memory”)	**AND**	(Rehabilitation OR Remediation OR Training OR Stimulation)	106
(“Parkinson's disease” OR “Parkinson disease”)	**AND**	(Memory)	**AND**	(Rehabilitation OR Remediation OR Training OR Stimulation)	451
(“Parkinson's disease” OR “Parkinson disease”)	**AND**	(“Executive function*”)	**AND**	(Rehabilitation OR Remediation OR Training OR Stimulation)	244

Note: The search strategy was filtered by title and abstract.
