# Supplementary material for: Effectiveness of Cognitive Rehabilitation in Parkinson’s Disease: A Systematic Review and Meta-Analysis
Source: J Pers Med. 2021 May 18;11(5):429. doi: 10.3390/jpm11050429 (PMC8157874; doi:10.3390/jpm11050429)
Supplement: Supplementary file 1 [file jpm-11-00429-s001.zip › SupplementaryMaterial_Table2_IbarretxeBilbao.pdf]

Supplementary material

Table S2: Methodological quality and risk of bias

ROB-2. Randomized studies							
**Study**	**PEDro Score**	**Bias arising from the randomization process**	**Bias due to deviation from intended interventions**	**Bias due to the missing outcome data**	**Bias in measurement of the outcome**	**Bias in selection of the reported result**	**Overall Risk of Bias**
**Sammer et al., 2006**	6	Unclear	Unclear	Low	High	Low	High
**París et al., 2011**	7	Unclear	Low	Unclear	Low	Low	Unclear
**Edwards et al., 2013**	8	Low	Low	Low	High	Low	High
**Cerasa et al., 2014**	8	Unclear	Low	Low	Low	Low	Unclear
**Costa et al., 2014**	8	Unclear	Low	Low	Low	Low	Unclear
**Peña et al., 2014**	8	Low	Low	Low	Low	Low	Low
**Petrelli et al., 2014**	8	Low	Low	Unclear	Low	Low	Unclear
**Angelucci et al., 2015**	7	Unclear	Low	Low	Low	Low	Unclear
**Fellman et al., 2018**	8	Low	Low	Low	Unclear	Low	Unclear
**Bernini et al., 2020**	8	Low	Low	Low	Low	Low	Low
**Ophey et al., 2020**	9	Low	Low	Low	Low	Low	Low

Table S2: Risk of bias (continued)

ROBINS-I. Non-randomized studies								
**Study**	**Bias due to confounding**	**Bias in selection of participants into the study**	**Bias in classification of interventions**	**Bias due to deviations from intended interventions**	**Bias due to missing data**	**Bias in measurement of outcomes**	**Bias in selection of the reported result**	**Overall Risk of Bias**
**Naismith et al., 2013**	Low	Low	Low	Low	Low	Low	Low	Low
