# Supplementary material for: Effectiveness of Cognitive Rehabilitation in Parkinson’s Disease: A Systematic Review and Meta-Analysis
Source: J Pers Med. 2021 May 18;11(5):429. doi: 10.3390/jpm11050429 (PMC8157874; doi:10.3390/jpm11050429)
Supplement: Supplementary file 1 [file jpm-11-00429-s001.zip › SupplementaryMaterial_Table3_IbarretxeBilbao.pdf]

Table S3: Outcomes and assessment included in the meta-analysis

**Global cognitive status**
Montreal Cognitive Assessment (MoCA)
Mini Mental State Examination (MMSE)
Demtec
Addenbrooke's Cognitive Examination (ACE)
**Attention**
Digit span forward
Brief test of attention (BTA)
Paced Auditory Serial Addition Test (PASAT)
**Working memory**
Face-Name-learning-Test (GNL)
Digit span backward
Demtec- Digit span reverse
**Verbal memory**
California Verbal Learning Test (CVLT)- Short and long delay free recall
Hopkins Verbal Learning Test- Revised (HVLT-R)– Short and long delay free recall
Selective Reminding Test (SRT)
Demtec- Verbal short and long-term memory
Memo Test- Verbal short and long-term memory
Verbal episodic memory- A sentence recall task, a word list recall task
Consortium to Establish a Registry of Alzheimer’s Disease Plus (CERAD +)- Wordlist Learning and recall
Logical Memory subtest of the Wechsler Memory Scale- III
**Visual memory**
Modified Taylor figure- delayed recall
Rey-Osterrieth Complex Figure Test (ROCFT)- Immediate and delayed recall
Brief Visuospatial Memory Test-Revised (BVMT-R)- Short and long delay free recall
**Verbal fluency**
The F-A-S Phonemic Verbal Fluency Test

Semantic- Animals
Demtec- Animals
Controlled Oral Word Association Test (COWAT)
Phonemic and Semantic Verbal Test
**Executive functions**
Behavioural Assessment of the Dysexecutive Syndrome (BADS)
Tower of London (ToL)
Stroop Color Word Interference - Words-Colors
Stroop Color Word Interference - Interference
Zoo Map
Trail Making Test- Part B (TMT-B)
**Visuospatial and visuoconstructive abilities**
Rey-Osterrieth Complex Figure Test (ROCFT)- copy
The Line Orientation subtest from the Repeatable Battery for the Assessment of Neuropsychological Status (RBANS)
Judgement of Line Orientation (JLO)
**Processing speed**
Symbol Digit Modalities Test (SDMT)
Trail Making Test- Part A (TMT-A)
The Salthouse Perceptual Comparison Test (SPCT)
Useful Field Of Vision (UFOV)
Stroop Color Word Interference- Word
Stroop Color Word Interference - Colors
**Depressive symptoms**
Geriatric Depression Scale (GDS-15)
Beck Depression Inventory II (BDI-II)
**Quality of life**
Parkinson's Disease Questionnaire (PDQ- 39)
